# Atomic and Molecular Structure Regulated In Situ Cross-Linked Polyurethane Gel Electrolyte for High-Performance Lithium Metal Batteries

**DOI:** 10.1007/s40820-026-02307-4

**Published:** 2026-08-03

**Authors:** Jialun Ni, Yong Zeng, De Ning, Xuan He, Xiaokang Ju, Xueling Liu, Rui Gao, Yingchun Xu, Ruijie Du, Dong Zhou, Jun Wang, Yongli Li

**Affiliations:** 1 Institute for Clean Energy Technology, North China Electric Power University, Beijing, 102206 People’s Republic of China; 2 School of Innovation and Entrepreneurship, Southern University of Science and Technology, Shenzhen, 518055 People’s Republic of China; 3 Centre for Photonics Information and Energy Materials, Shenzhen Institutes of Advanced Technology, Chinese Academy of Sciences, Shenzhen, 518055 People’s Republic of China; 4 Sunwoda Mobility Energy Technology Co., Ltd, Shenzhen, 518107 People’s Republic of China; 5 School of Advanced Energy, Sun Yat-Sen University Shenzhen Campus, Shenzhen, 518107 People’s Republic of China

**Keywords:** In situ polymerization, Lithium metal batteries, Polyurethane electrolytes, Solvation structure

## Abstract

The incompatibility of conventional electrolytes with high-voltage cathodes and lithium metal anodes limits the performance of lithium metal batteries (LMBs). Here, an in situ cross-linked polyurethane gel electrolyte (G-P3 AR) is designed through atomic and molecular structure regulation. The polyester segments widen the highest occupied molecular orbital–lowest unoccupied molecular orbital gap, extending the electrochemical stability window to 4.97 V for compatibility with NCM811 cathodes. Polyether segments exhibit a lower Li^+^ binding energy, reducing the desolvation barrier and enhancing anode stability. At the atomic level, *sp*^2^-hybridized boron in the chain extender immobilizes anions (TFSI^−^ and DFOB^−^) through Lewis acid–base interactions, raising the Li^+^ transference number to 0.78 and enabling exceptional rate capability (157.7 mAh g^−1^ at 2 C in the Li||NCM811 cell). Hydrogen bonding between the polymer and solvent restructures the solvation sheath, promoting inorganic-rich interphases. The Li|G-P3 AR|NCM811 cell retains 81.7% capacity after 500 cycles at 0.5 C charge/1 C discharge, demonstrating a rational electrolyte design strategy for high-performance LMBs.

**Supplementary Information:**

The online version contains supplementary material available at 10.1007/s40820-026-02307-4.

## Highlights

- Hybrid polyester/polyether segments simultaneously enable high-voltage stability (4.97 V) and fast Li^+^ desolvation in Li||NCM811 batteries for both cathode and anode compatibility.
- The *sp*^2^-hybridized boron Lewis acid centers anchor anions via acid-base interactions, boosting Li^+^ transference number to 0.78 and enhancing rate capability (157.7 mAh g^−1^ at 2 C).
- Hydrogen bonding restructures solvation sheath, promoting anion-derived inorganic-rich solid electrolyte interphase/cathode electrolyte interphase that delivers 81.7% capacity retention after 500 cycles.

**Supplementary Information:**

The online version contains supplementary material available at 10.1007/s40820-026-02307-4.

## Graphical abstract

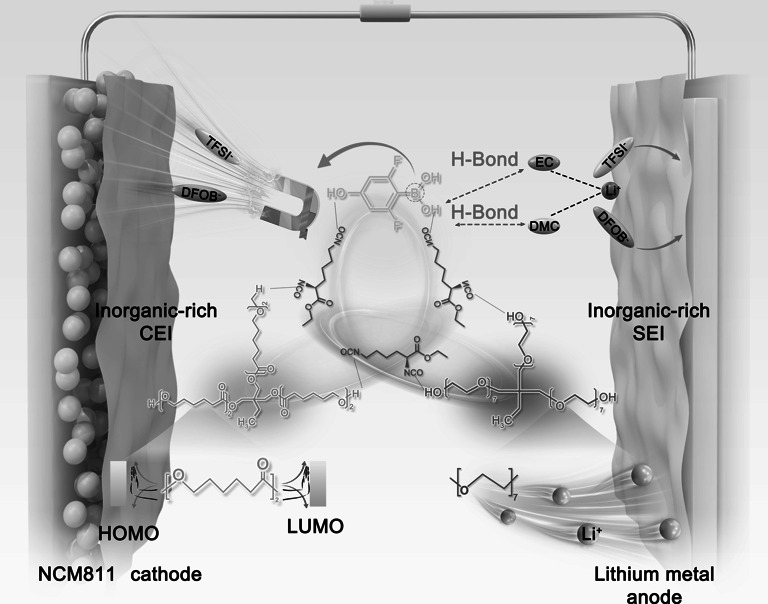

## Introduction

The escalating global demand for high-energy–density storage systems, propelled by the rapid adoption of clean energy technologies and the explosive growth of the electric vehicle market, has starkly revealed the limitations of conventional lithium-ion batteries [1, 2]. The ubiquitous “rocking-chair” systems, typically based on LiCoO_2_ or LiFePO_4_ cathodes paired with graphite anodes, are approaching their theoretical energy density ceiling [3, 4]. This inherent limitation has catalyzed intense research into next-generation battery chemistries, among which lithium metal batteries (LMBs) stand out as a particularly promising candidate. The appeal of LMBs lies in the use of metallic lithium as the anode, which boasts an ultrahigh theoretical capacity (3860 mAh g^−1^) and the lowest electrochemical potential (− 3.04 V vs. SHE) among all anode materials [5–7]. A pivotal strategy to realize the full potential of LMBs is to couple the high-capacity lithium metal anodes (LMAs) with high-voltage Ni-rich layered oxide cathodes, such as LiNi_0.8_Co_0.1_Mn_0.1_O_2_ (NCM811) [8, 9]. However, the practical implementation of this promising Li||NCM811 configuration is severely hampered by the intrinsic inadequacies of conventional carbonate-based liquid electrolytes, which fail to simultaneously maintain good compatibilities with both the highly reductive LMAs and the oxidizing NCM811 cathodes at high voltage [10, 11].

The core of the electrochemical stability for the Li||NCM811 LMBs is a fundamental trade-off in electrolyte chemistry. Ether-based electrolytes, known for their good compatibility with LMA and ability to form a relatively stable solid electrolyte interphase (SEI), exhibit a narrow electrochemical stability window (ESW, typically below 4 V vs. Li/Li^+^) [12]. This leads to their irreversible oxidative decomposition at the operating voltages of Ni-rich cathodes (≥ 4.3 V) with a thick cathode electrolyte interphase (CEI), resulting in rapid capacity fade [13]. Regarding the ester-based electrolytes (e.g., carbonates), they usually offer a wider ESW, making them ostensibly suitable for high-voltage operation. However, their strong solvation with Li^+^, driven by the highly electronegative carbonyl groups (C=O), creates a high desolvation energy barrier at the anode interface, hindering Li^+^ transport kinetics [14, 15]. Furthermore, the decomposition of these strongly coordinated solvent molecules tends to form an SEI rich in organic components (e.g., ROCO_2_Li), which is mechanically fragile and incapable of uniformly regulating Li^+^ deposition or suppressing lithium dendrite growth [16]. Researchers have explored various strategies to overcome these challenges, such as fluorinated ether solvents [17, 18] and high-concentration electrolytes (HCEs) [19, 20], but these often introduce new problems like high viscosity [21], poor wettability and low Li^+^ transference number (*t*_*Li*+_) [22], underscoring the need for a more holistic and systematic electrolyte design paradigm.

In recent years, in situ polymerized gel polymer electrolytes (GPEs) have emerged as a transformative approach to break these trade-offs [23]. By forming a solid polymer network in situ within the liquid electrolyte, GPEs combine the high ionic conductivity of liquids with the enhanced mechanical stability and interfacial compatibility of solids [24–26]. The polymer network can effectively regulate solvent mobility [27, 28] and influence ion coordination environments [29, 30]. Recent advances in this field have explored diverse strategies for tailoring solvation structures, ranging from covalently integrating anions into polymer backbones [31] to employing fluorinated monomers that create weakly solvating domains [32]. While these approaches have demonstrated impressive performance, they often rely on bespoke monomer design, which may complicate synthesis and scalability. Alternatively, leveraging commercially available polymers offers a complementary and more accessible pathway to achieve comparable solvation regulation. Among various polymers, polyurethanes have garnered significant attention as a particularly versatile matrix for advanced electrolytes. As a class of block copolymers, polyurethanes possess a unique microphase-separated structure comprising “soft” segments (often polyethers or polyesters) that facilitate ion transport and “hard” segments that provide mechanical integrity [9, 33, 34]. Crucially, the chemical composition and ratio of these segments can be precisely tailored, allowing for multi-dimensional customization of properties such as the electronic structure, functional group density, intermolecular forces and the spatial configuration of the cross-linked network. This inherent structural tunability positions polyurethane as an ideal matrix for designing electrolytes that can simultaneously address the multifaceted challenges of LMBs. However, existing polyurethane-based electrolytes predominantly rely on single-type soft segments (either polyether or polyester), inevitably inheriting the intrinsic trade-offs of conventional liquid electrolytes and failing to simultaneously achieve high-voltage tolerance and favorable lithium metal compatibility. Moreover, precise atomic-scale regulation within cross-linked polyurethane networks remains largely unexplored. Consequently, synergistically integrating ether/ester hybrid segments with functional chain extenders to overcome these trade-offs warrants further investigation.

**Fig. 1:**
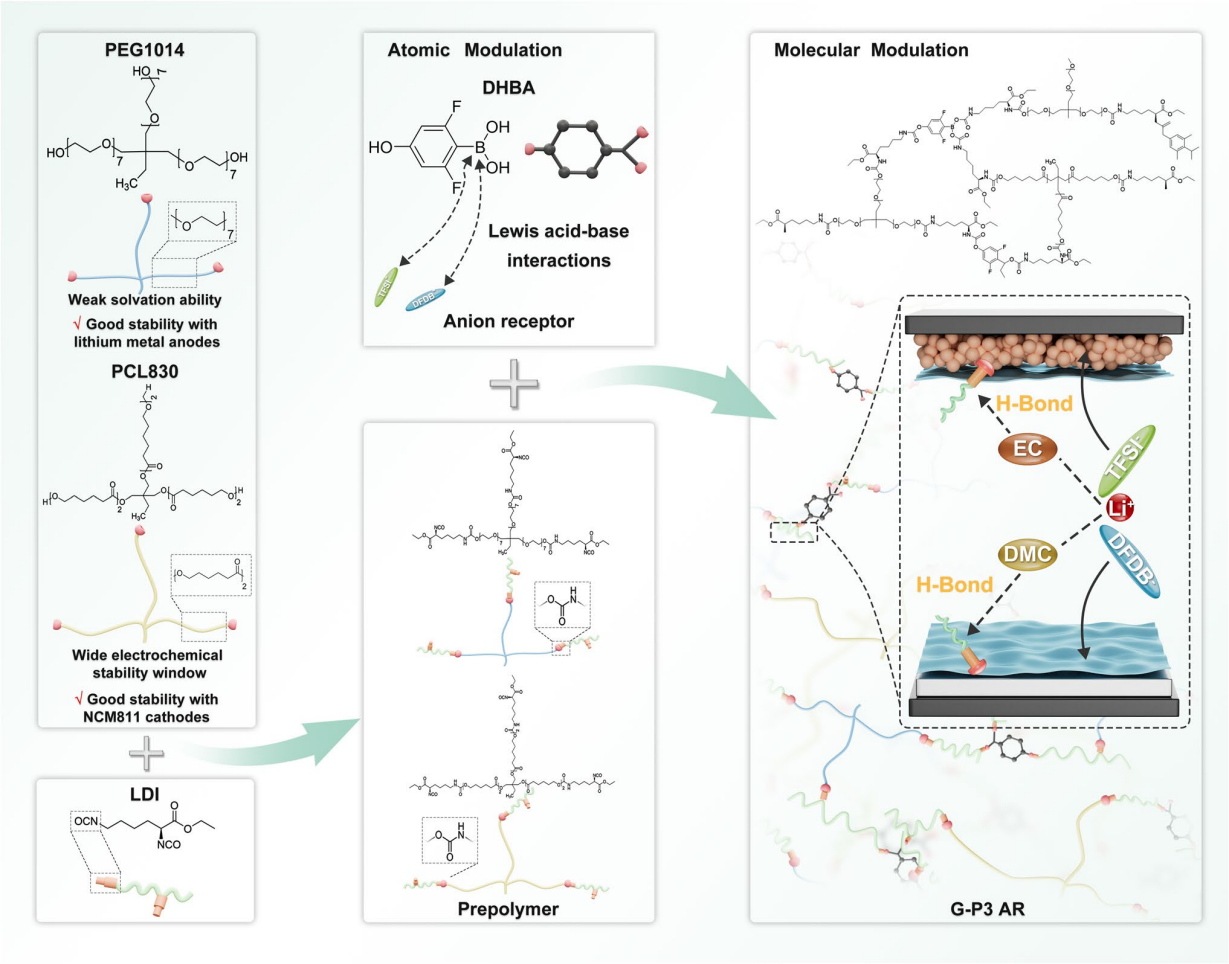
Schematic illustrations of atomic and molecular structure regulation design concept and working mechanism

In this study, we proposed an integrated atomic and molecular electronic structure regulation strategy embedded within an in situ polymerized polyurethane GPE (G-P3 AR) to systematically overcome the inherent limitations of conventional electrolytes. As shown in Fig. 1, the incorporated polyester segments into the polymer backbone widen the highest occupied molecular orbital (HOMO)–lowest unoccupied molecular orbital (LUMO) gap to significantly expand the ESW and ensure good compatibility with high-voltage NCM811 cathodes. Meanwhile, polyether segments exhibit a relatively weaker Li^+^ binding affinity compared to polyester segments, facilitating Li^+^ release from the polymer chain at the electrode interface, thereby enhancing anode stability. Specially, the introduction of chain extender featuring with the electron-deficient and *sp*^2^-hybridized boron atom acts as a Lewis acid, effectively anchoring anions from lithium salt (TFSI^−^ and DFOB^−^) through Lewis acid–base interactions. As a result, the *t*_*Li*+_ is dramatically increased to 0.78 and the polarization is noticeably suppressed during high-rate cycling. Moreover, we leveraged the abundant hydrogen bonding capability of the urethane linkages (–NH–COO–) in the polymer backbone. These groups interact with solvent molecules (e.g., EC, DMC), redistributing their electron cloud and reducing their binding energy with Li^+^. This reconstruction of the solvation structure facilitates Li^+^ desolvation and, more importantly, promotes participation of anions in the primary solvation sheath, leading to the formation of inorganic-rich SEI and CEI layers. When deployed the G-P3 AR in Li||NCM811 cells, it enables outstanding long-cycle stability, with 81.7% capacity retention after 500 cycles at 1C. This work not only presents a novel electrolyte for high-performance LMBs but also establishes a new paradigm for the dual-scale rational design of advanced electrolytes, offering fundamental insights into the intricate relationship between molecular structure and electrochemical performance.

## Experimental Section

### Materials

Ethylene carbonate (EC), dimethyl carbonate (DMC), lithium bis (trifluoromethanesulfonyl) imide (LiTFSI, 99.95%) and lithium difluoro(oxalato)borate (LiDFOB, 99.95%) were purchased from DoDochem Co., Ltd. Trimethylolpropane ethoxylate (PEG1014, *M*_*w*_ = 1014), polycaprolactone triol (PCL830, *M*_*w*_ = 830), L-lysine diisocyanate (LDI, 95%), (2,6-difluoro-4-hydroxyphenyl)boronic acid (DHBA, 97%) and dibutyltin dilaurate (DBTDL, 95%) were sourced from Shanghai Macklin Biochemical Technology Co., Ltd. LiNi_0.8_Co_0.1_Mn_0.1_O_2_ (NCM811), aluminum foil (18 μm, 99.3%) and copper foil (9 μm, 99.3%) were supplied from Kejing Star Technology Corp. Super P (99.9%), polyvinylidene difluoride (PVDF), Li foils (15.6 mm diameter disk, 99.95%) were purchased from Guangdong Canrd New Energy Technology Co., Ltd.

### Preparation of GPEs

Liquid electrolyte (LE) was prepared by dissolving 2 M LiTFSI and 0.2 M LiDFOB in a mixture of EC and DMC with a volume ratio of 3:7. G-P(EG-U): PEG1014 was dissolved in LE under stirring until a clear solution formed. LDI was then added at a molar ratio of PEG1014 to LDI of 2:3, with the combined weight of both components accounting for 15 wt% of the total electrolyte mass. After stirring for 30 min at room temperature, the catalyst (0.5 wt% DBTDL) was introduced. The resulting homogeneous solution was injected into either coin-type cells (50 µL) or pouch cells (3.5 g Ah^−1^) and sealed. In situ polymerization was conducted by heating the cells at 45 °C for 10 h to form GPEs. All procedures, including electrolyte preparation and cell assembly, were performed in an argon-filled glove box (Mikrouna; O_2_ < 0.01 ppm, H_2_O < 0.01 ppm) under ultrapure argon (≥ 99.99%, Air Products).

G-P(CL-U): PCL830 was dissolved in LE under stirring until a clear solution formed. LDI was then added at a molar ratio of PCL830 to LDI of 2:3, with the combined weight of both components accounting for 15 wt% of the total electrolyte mass. Subsequent procedures followed the same protocol as above.

G-P3: PEG1014 and PCL830 (1:1 molar ratio) were dissolved in LE under stirring until a clear solution formed. LDI was then added at a molar ratio of PEG 1014 to LDI of 1:3. Subsequent procedures followed the same protocol as above.

G-P(CL-U) AR: PCL830 was dissolved in LE under stirring until a clear solution formed. LDI was then added at a molar ratio of PCL830 to LDI of 1:3, followed by polymerization at 45 °C for 2 h after thorough mixing. DHBA was then introduced at a PCL830: DHBA molar ratio of 1:1. After dissolution via stirring, the catalyst was added to form the precursor solution. Subsequent procedures followed the same protocol as above.

G-P(EG-U) AR: The preparation procedure was identical to that of G-P(CL-U) AR, except that PCL830 was replaced by PEG1014.

G-P3 AR: PEG1014 and PCL830 (1:1 molar ratio) were dissolved in LE under stirring until a clear solution formed. LDI was then added at a molar ratio of PEG1014 to LDI of 1:6, followed by polymerization at 45 °C for 2 h after thorough mixing. DHBA was then introduced at a PEG1014: DHBA molar ratio of 1:2. After dissolution via stirring, the catalyst was added to form the precursor solution. Subsequent procedures followed the same protocol as above.

### Preparation of P3 and P3 AR

The required reagents were added to DMC according to the above procedures. After thermal polymerization, the mixture was further heated at 60 °C for 12 h to remove DMC.

### Electrochemical Measurements

Li symmetric cells were constructed in CR2025 coin cells with Li foils and electrolyte. The Li||Cu cells utilized a Li foil (550 μm thick, 15.6 mm diameter), a Celgard2500 separator (25 μm thick, 18 mm diameter), and a Cu foil (10 μm thick, 14 mm diameter). The NCM811 cathodes were fabricated following a slurry process: 80 wt% active material, 10 wt% PVDF, and 10 wt% Super P were mixed in NMP, coated onto an Al foil, dried at 100 °C under vacuum for 12 h, and cut into 12 mm disks. Two cathodes with areal loadings of 3 and 10 mg cm^−2^ were prepared. The pouch cell was assembled using an NCM811 cathode (areal loading: 10 mg cm^−2^; areal capacity: 2.2 mAh cm^−2^; dimensions: 5 × 3 cm^2^) paired with a 50 μm lithium foil anode (areal capacity: 9.65 mAh cm^−2^; dimensions: 5.2 × 3.2 cm^2^), giving an N/P ratio of 4.8.

The *Coulombic* efficiency (CE) measurement for Li||Cu cells utilized the Li foil as the source, with its total charge defined as *Q*_Li_. From this reservoir, a specific charge amount (*Q*_*c*_) was allocated for plating/stripping cycles between the electrodes. Following *n* cycles, the residual Li in the electrode was fully stripped to the cutoff voltage. By measuring the amount of Li remaining after cycling, the average CE (ACE) over *n* cycles can be calculated by Eq. (1):

1 $${\mathrm{CE}} = \frac{{nQ_{c} + Q_{{\mathrm{s}}} }}{{nQ_{c} + Q_{{{\mathrm{Li}}}} }} \times 100\%$$

where *Q*_S_ corresponds to the final stripped charge or the amount of Li remaining after *n* cycles.

The ionic conductivity of the electrolyte was measured through electrochemical impedance spectroscopy (EIS) with an amplitude of 10 mV from 1 MHz to 0.1 Hz in the symmetric battery of stainless steel (SS)||SS. The ionic conductivity can be calculated using the following Eq. (2):

2 $$\sigma =\frac{L}{RS}$$

where *L* is the thickness of the separator, *S* is the contact area, and *R* is resistance.

To evaluate the ESW of electrolytes, linear sweep voltammetry (LSV) tests were conducted on Li||SS cells over a voltage range of 2–6 V at a scan rate of 0.1 mV s^−1^.

Activation energy (*E*_a_) for ionic conduction in electrolytes was acquired from the time-dependent ionic conductivity in a temperature range of 25–65 °C and calculated based on Eq. (3):

3 $${\sigma}_{\mathrm{T}}T=A\mathrm{e}\mathrm{x}\mathrm{p}(\frac{-{E}_{\mathrm{a}}}{RT})$$

where *σ*_T_ is the ionic conductivity at different temperatures, *R* is the gas constant, *T* represents temperature, and A corresponds to the pre-exponential factor [35].

The *t*_Li+_ was measured by time-varying measurements with an applied 10 mV polarization voltage for 6000 s and calculated by the following Eq. (4):

4 $${t}_{\mathrm{L}\mathrm{i}}^{+}=\frac{{I}_{s}\left(\Delta V-{R}_{0}{I}_{0}\right)}{{I}_{0}\left(\Delta V-{R}_{s}{I}_{\mathrm{s}}\right)}$$

Here, *R*_*0*_ and *R*_*s*_ denote the interfacial resistance of the lithium electrode before and after polarization, respectively. *I*_*0*_ is the current at the start of the polarization, while *I*_s_ refers to the steady-state current through the electrode during the polarization. *ΔV* is the polarization potential (10 mV).

## Results and Discussion

### Molecular Structure Design and Electrochemical Properties

Ether-based and ester-based electrolytes exhibit complementary advantages on LMAs and high-voltage cathodes. However, simple physical mixing may lead to crosstalk and induce a shortcoming effect [36]. Based on these considerations, we introduced polyether triol (PEG1014) and polyester triol (PCL830) into a single polyurethane molecule via a simple addition reaction. This design aims to enable G-P3 AR to achieve a wide ESW along with a relatively weak solvating ability. In addition, a *sp*^2^-hybridized boron atom from DHBA was introduced as an anion receptor, which utilizes its electron-deficient nature to coordinate anions and accelerate Li^+^ transport. On one hand, this chain extender design enables polymer molecules to exhibit lower crystallinity. On the other hand, the more abundant hydrogen bonding interactions between polymer and solvent molecules reconstruct the solvation structure, inducing inorganic-rich SEI/CEI layers on the electrode surface. This unique design establishes a dual-scale enhancement mechanism, with the characteristic urethane groups of polyurethane serving as the structural bridge to integrate these three key components, unifying their distinct advantages within a single polymer matrix for optimized performance.

**Fig. 2:**
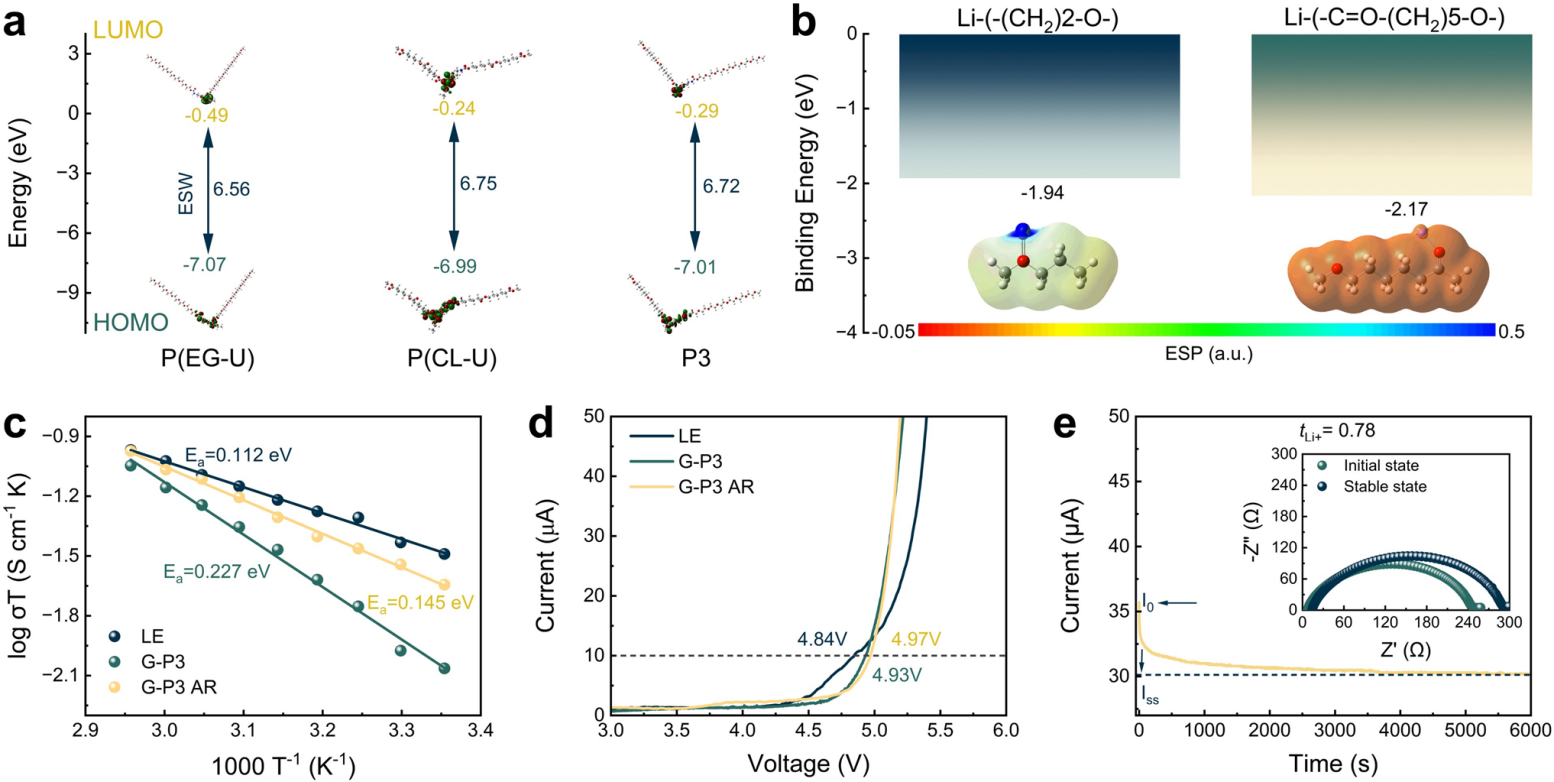
Dual-scale regulation enhancing electrolyte performance. **a** Energy levels of the frontier orbitals for the characteristic fragment structures in P(EG-U), P(CL-U) and P3. **b** Li^+^ binding energies with PEG1014 and PCL830 repeating units. **c** *Arrhenius* plots of different electrolytes. **d** LSV curves of different electrolytes. **e** Polarization curves and the *Nyquist* plots before and after polarization of Li|G-P3 AR|Li

Density functional theory (DFT) simulations were performed to calculate the HOMO and LUMO energies of characteristic fragments in polyurethanes synthesized with different polymer triols as soft segments. The HOMO–LUMO energy gap directly governs the ESW, thereby determining the maximum voltage tolerance of the electrolyte during cycling [37, 38]. As illustrated in Fig. 2a, the HOMO–LUMO energy levels of characteristic segments from three polyurethane compositions are calculated: (i) P(EG-U) (polyether-based soft segment), (ii) P(CL-U) (polyester-based soft segment) and (iii) P(EG-CL-U) (abbreviated as P3, hybrid soft segment with polyether and polyester at a 1:1 molar ratio). The results show that the HOMO–LUMO gap is 6.56 eV for P(EG-U), while P(CL-U) exhibits the widest gap of 6.75 eV. Under the influence of polyether segments, P3 shows a slightly reduced gap of 6.72 eV. The incorporation of polyester segments thus broadens the ESW of the polymer matrix, stabilizing GPEs under the operating voltages of NCM811 cathodes. Furthermore, Fig. 2b compares the binding energy between Li^+^ and one repeating unit of PEG1014 or PCL830 (i.e.,–(CH_2_)_2_–O– for PEG1014 and –C=O–(CH_2_)_5_–O– for PCL830). Compared to PCL830 (− 2.17 eV), PEG1014 exhibits a relatively lower Li^+^ binding energy (− 1.94 eV), which reduces the desolvation energy barrier and facilitates more favorable desolvation kinetics at the lithium metal interface [39]. Additionally, electron density distribution analysis of the DHBA-centered polyurethane segment (Fig. S1a) confirms that the *sp*^2^-hybridized boron atom carries a positive charge, and DFT-calculated binding energies of − 0.36 eV for B-TFSI^−^ (Fig. S1b) and − 0.41 eV for B-DFOB⁻ (Fig. S1c) interactions confirm its role as a Lewis acid site for anion anchoring.

To investigate the effects of soft segments and chain extender on electrochemical properties, four GPEs (G-P(EG-U), G-P(CL-U), G-P3, and G-P3 AR) with different chemical compositions were synthesized via the polyaddition reaction between hydroxyl groups (–OH) of polymer triols and isocyanate groups (–NCO) of LDI to form urethane linkages (–NH–COO–). After thermal polymerization, the initially liquid precursor was converted into a transparent solid product (Fig. S2a, b). Fourier transform infrared (FTIR) spectroscopy was employed to confirm complete polymerization. As shown in Fig. S2c, original PEG1014, PCL830 and DHBA samples exhibit a broad absorption peak at 3450 cm^−1^, attributed to the –OH stretching vibration [40]. The characteristic absorption band at 2258 cm^−1^ corresponds to the asymmetric stretching vibration of the –NCO in LDI. In contrast, the FTIR spectra of the thermally polymerized G-P3 and G-P3 AR show the complete disappearance of –OH and –NCO absorption bands. Simultaneously, two new bands emerge in Fig. S2d: 1526 cm^−1^ (assigned to –C–N–H– bending vibration) and 3312 cm^−1^ (assigned to hydrogen-bonded –NH– stretching vibration) [33]. These spectral changes collectively confirm the successful synthesis of the polyurethane structure. Precursors without DBTDL remain liquid after heating (Fig. S3a, b) and show no –C–N– peak by FTIR spectra (Fig. S3c), indicating incomplete reaction without the catalyst.

The activation energies of LE, the G-P3 and G-P3 AR were determined via temperature-dependent conductivity measurements (25–65 °C) following *Arrhenius* behavior (Fig. S4). The G-P3 AR shows a Li^+^ conductivity of 0.78 mS cm^−1^ at 25 °C with an activation energy of 0.145 eV, while the LE and G-P3 exhibit activation energies of 0.115 and 0.227 eV, respectively (Fig. 2c). These results indicate that incorporating a chain extender enables the GPE to achieve an activation energy comparable to that of LE. This phenomenon may be attributed to the chain extender, which introduces additional branching points into the main chain, further increasing the disorder of the polyurethane cross-linked network. The crystallinity of the polyurethane was verified using X-ray diffraction. As shown in Fig. S5a, the P3 AR exhibits a broad and diffuse peak, indicating an amorphous structure [41]. Additionally, the lower glass transition temperature (*T*_g_) of the polymer component P3 AR in G-P3 AR (− 39.3 °C, compared to − 34 °C for P3 in G-P3) is indicative of enhanced polymer chain mobility and increased free volume, which may facilitate ion migration pathways (Fig. S5b) [42]. Consequently, the G-P3 AR demonstrates higher ionic conductivity.

As shown in the LSV measurement in Fig. S6, the G-P(EG-U) displays the narrowest ESW, which is in good agreement with DFT results. As shown in Fig. 2d, the G-P3 AR exhibits an extended ESW of up to 4.97 V. The enhancement over the G-P3 (4.93 V) is attributed to the electron-withdrawing C–F groups in the DHBA, which effectively lower the electron density around the oxygen atoms, making them less susceptible to oxidation [43, 44]. EIS measurements were performed to determine the *t*_Li+_ of different electrolytes. Critically, the *t*_*Li*+_ increases from 0.44 for the LE (Fig. S7a) and 0.55 for the G-P3 (Fig. S7b) to 0.78 for the G-P3 AR (Fig. 2e). This superior enhancement substantially mitigates concentration polarization during cycling, resulting in improved rate capability and prolonging cycle life in high-voltage applications [45, 46]. Moreover, the increased *t*_Li+_ directly validates the *sp*^2^-hybridized boron in DHBA as an efficient anion acceptor.

### Modulating Solvation Structure of Electrolytes

**Fig. 3:**
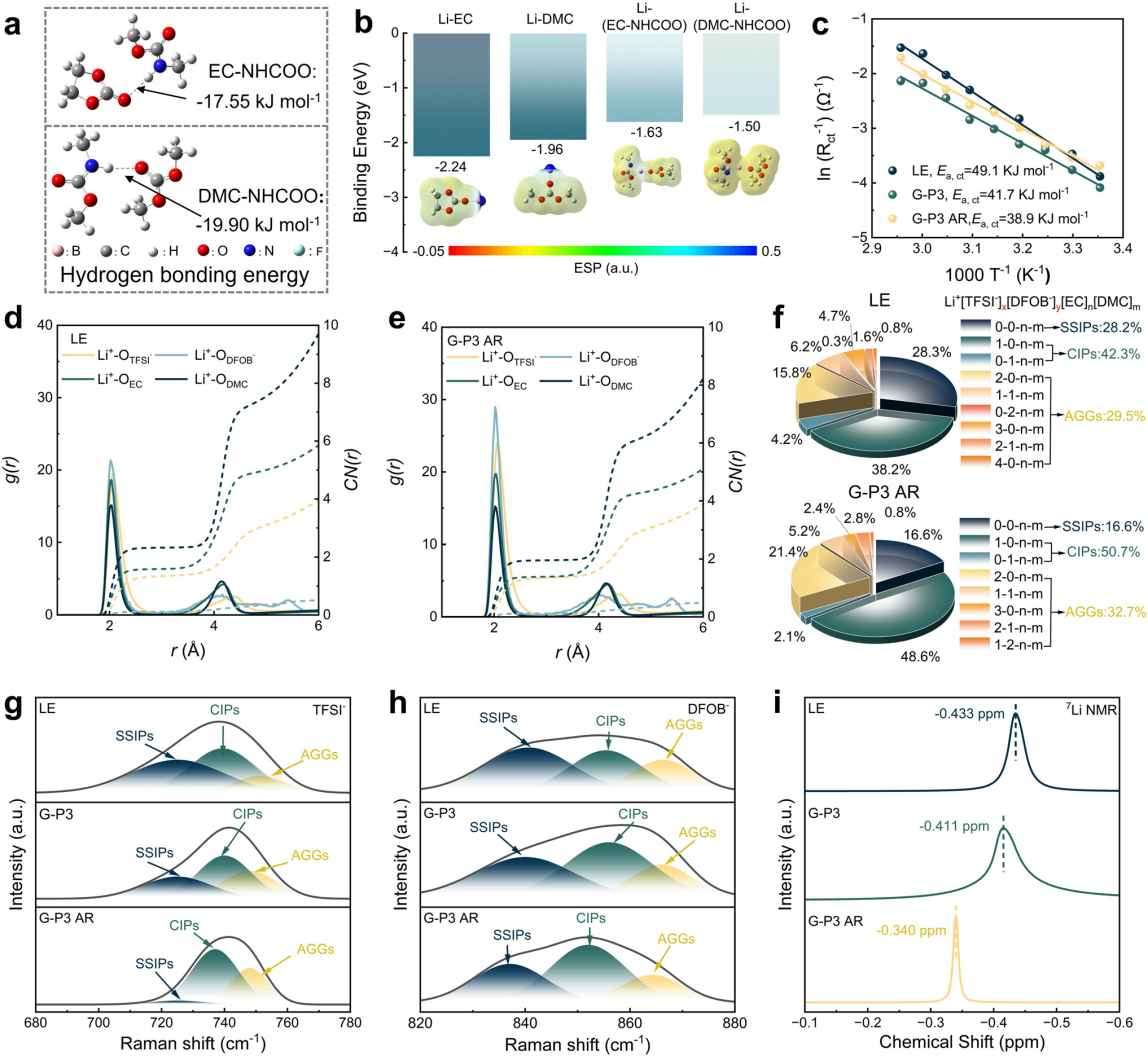
Intermolecular hydrogen bonding interactions restructuring Li^+^ solvation structure. **a** Hydrogen bonding interactions between polymers and solvents. **b** Li^+^ binding energies with original and hydrogen-bond-regulated solvents. **c** Activation energies for Li^+^ desolvation process in LE, G-P3 and G-P3 AR. RDF *g*(*r*) and coordination numbers *n*(*r*) calculated through MD simulations for **d** LE and **e** G-P3 AR. **f** Percentages of solvation structures in LE and G-P3 AR from MD simulation. Raman spectra of **g** TFSI^−^ and **h** DFOB^−^ in different electrolytes. **i** ^7^Li NMR spectra of different electrolytes

The solvation structure of Li^+^ plays a critical role in determining ion transport kinetics and interfacial stability. In the GPEs, the restructuring of the Li^+^ solvation environment arises from the synergistic interplay between direct chelation and intermolecular hydrogen bonding. The polymer backbone contains multiple Lewis basic sites, including ether oxygen atoms in polyether segments and carbonyl oxygen atoms in urethane linkages and polyester segments, which competitively coordinate with Li^+^ against EC/DMC solvent molecules, thereby weakening Li^+^–solvent binding [47, 48]. Concurrently, hydrogen bonding interactions between the polymer backbone and solvent molecules further modulate Li^+^ coordination environments [49, 50]. Specifically, the –NH groups in the polymer chains act as hydrogen bond donors, while solvent molecules (EC, DMC) serve as hydrogen bond acceptors. This interaction redistributes the solvent electron cloud, reducing their most negative electrostatic potential (*ESP*_*min*_), thereby weakening Li^+^–solvent coordination and facilitating anion participation in Li^+^ conduction. Given that the characteristic –NH–COO– groups in polyurethanes serve as both hydrogen bond acceptors and donors, extensive interchain hydrogen bonding exists within the polymer matrix. The hydrogen bonding energies between the –NH–COO– group and EC/DMC were quantified via DFT calculations (Fig. 3a), revealing favorable interactions between the –NH donor and the carbonyl oxygen acceptors of the solvent molecules. This computational result demonstrates the intrinsic hydrogen-bonding donor capacity of –NH toward external carbonyl oxygens. Experimentally, FTIR spectroscopy was performed on the dry polymer matrices and the corresponding gel electrolytes to probe how solvent molecules interact with the hydrogen-bonded polymer network. In the P3 AR, the N–H stretching vibration appears at 3336.8 cm^−1^, reflecting extensive interchain hydrogen bonds. Upon swelling with the liquid electrolyte, this band shifts to 3392.8 cm^−1^ in the G-P3 AR (Fig. S8a). A similar trend is observed for the P3 system, where the N–H peak shifts from 3350.3 to 3389.4 cm^−1^ upon gelation (Fig. S8b). Notably, the blue shift is substantially larger for G-P3 AR (56.0 cm^−1^) than for G-P3 (39.1 cm^−1^), because DHBA increases the –NH group density at equal mass thereby strengthening polymer–solvent interactions and disrupting a greater fraction of the original interchain hydrogen bonding network [51]. The competitive disruption of interchain hydrogen bonds by penetrating solvent molecules, coupled with the immobilization of solvent carbonyls via new hydrogen bonding interactions, attenuates the solvating ability of solvent molecules. Furthermore, as shown in Fig. 3b, the binding energies between Li^+^ and the original solvent molecules versus those regulated by hydrogen bonding reveal a significant reduction from − 2.63 to − 1.63 eV in the hydrogen-bond-regulated EC and − 1.96 to − 1.50 eV in the hydrogen-bond-regulated DMC. This marked attenuation quantitatively confirms the weakened solvation ability of solvent molecules. The interactions optimize electrolyte performance through two key pathways: firstly, they reconstruct the solvation structure by reducing the binding strength between solvent molecules and Li^+^, thereby facilitating the Li^+^ desolvation process; secondly, the weakened solvent solvation enables anions to access the primary solvation sheath more readily, promoting anion-derived decomposition reactions that contribute to the formation of inorganic-rich SEI/CEI layers [52, 53]. To quantify the Li^+^ desolvation capability, temperature-dependent EIS measurements were performed on Li||Li symmetric cells (Fig. 3c). The calculations based on the *Arrhenius* equation show that the Li^+^ desolvation activation energy (*E*_a, ct_) of the G-P3 AR is 38.9 kJ mol^−1^, which is significantly lower than those of the other systems (LE: *E*_a, ct_ = 49.1 kJ mol^−1^; G-P3: *E*_a, ct_ = 41.7 kJ mol^−1^). These values demonstrate that the abundant hydrogen bond donors in the G-P3 AR facilitate desolvation and endow Li^+^ with fast transport kinetics.

Molecular dynamics (MD) simulations were conducted to characterize Li^+^ migration dynamics in LE and G-P3 AR, revealing profound structural rearrangements driven by this binding preference. Radial distribution function (RDF) and coordination number *n*(r) analyses quantitatively confirm the solvation structure transition in the G-P3 AR: the *n*(r) of Li^+^–O (EC/DMC) drastically decreases due to hydrogen bonding, indicating the partial displacement of solvents. The suppressed solvent coordination reduces parasitic reduction reactions and mitigates solvent decomposition. Concurrently, the RDF analysis validates anion-dominated coordination dynamics, where the surge in Li^+^–O (TFSI^−^/DFOB^−^) peak intensity confirms preferential Li^+^–anion binding over solvent interactions (Fig. 3d, e). Moreover, based on the statistics of the number of ions/solvent molecules coordinated to Li^+^, the percentages of solvation structures for the LE and G-P3 AR were determined (Fig. 3f). Solvation structures with zero anions in the Li^+^ solvation sheath are defined as SSIPs, those with one anion as CIPs, and those with two or more anions as AGGs [54]. In the G-P3 AR, the proportion of SSIPs (16.6%) is much lower than that in the LE (28.2%), replaced by higher proportions of CIPs and AGGs. This change significantly promotes anion-derived decomposition, which is critical for forming robust SEI/CEI layers.

As shown in Fig. 3g, h, the introduction of in situ polyurethane significantly reduces the SSIPs’ proportion as validated by Raman spectroscopy. This indicates that hydrogen bonding interactions effectively promote the broader participation of anions (TFSI^−^ or DFOB^−^) in the Li^+^ solvation sheath, leading to an increased proportion of CIPs and AGGs. ^7^Li nuclear magnetic resonance (NMR) further validates this solvation restructuring (Fig. 3i). Compared to the LE, the G-P3 exhibits a downfield shift to − 0.411 ppm, while the G-P3 AR shows a more pronounced shift to − 0.340 ppm, indicating reduced electron density around Li^+^ due to enhanced anion coordination [55, 56]. Notably, the G-P3 AR performs better than the G-P3, which is attributed to the introduction of the chain extender, resulting in more hydrogen bonds within the polymer network at the same mass fraction in the GPEs.

### Electrochemical Performances in Different Cell Configurations

**Fig. 4:**
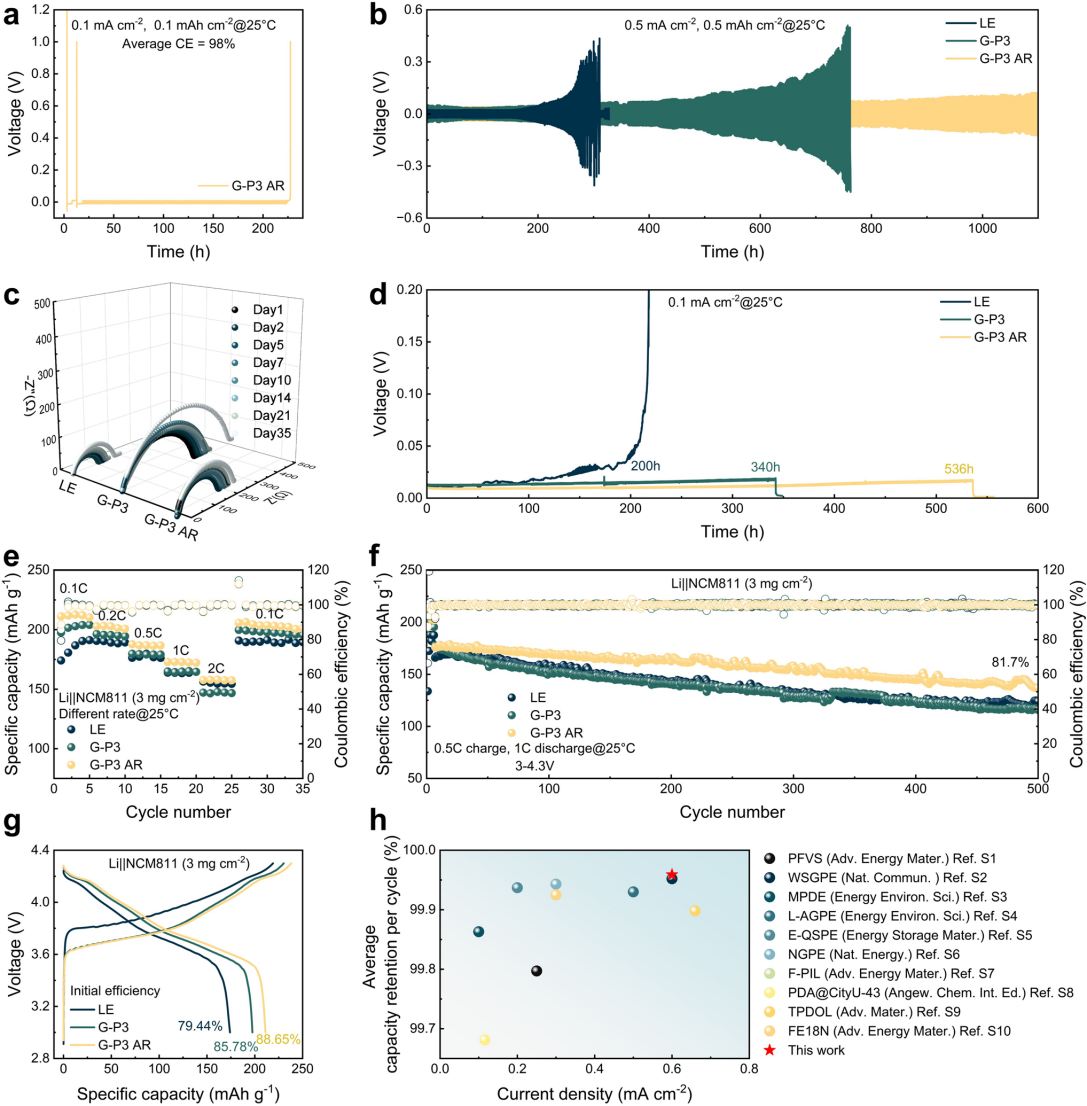
Comprehensive electrochemical superiority across multiple cell configurations. **a** Voltage curves of Li|G-P3 AR|Cu cell. **b** Galvanostatic cycling curves of Li symmetric cells with different electrolytes at 0.5 mA cm^−2^, 0.5 mAh cm^−2^ @25 °C. **c** *Nyquist* plots of Li||Li symmetric cells with different electrolytes after different days. **d** Galvanostatic polarization curves of Li||Li symmetric cells with different electrolytes at a current density of 0.1 mA cm^−2^. **e** Rate capabilities of Li||NCM811 cells with different electrolytes. **f** Cycling performance of Li||NCM811 cells with different electrolytes at 0.5 C charge/1 C discharge. **g** Galvanostatic charge/discharge curves of Li||NCM811 cells with different electrolytes at the first cycle. **h** Comparison of different GPEs/QSEs in terms of discharge current density and average capacity retention per cycle with literature data

The Li plating/stripping CE was evaluated in Li||Cu cells using the method reported by Adams et al. [57]. When cycled at 0.1 mA cm^−2^, 0.1 mAh cm^−2^ for 100 cycles, the Li|G-P3 AR|Cu cell achieves an ACE of 98% with a low active lithium loss rate of 0.02% per cycle (Fig. 4a), outperforming that of the Li|G-P3|Cu cell (ACE of 97%, 0.03% loss per cycle; Fig. S9b). In stark contrast, the Li|LE|Cu cell exhibits a sudden voltage surge (> 1 V vs*.* Li^+^/Li) after 50 cycles (Fig. S9a), indicating complete depletion of the pre-deposited lithium reservoir due to the continuous formation of SEI. The lithium plating/stripping performance of lithium symmetric cells was further evaluated to assess the interfacial stability between these electrolytes and LMAs. As shown in Fig. 4b, the Li|G-P3 AR|Li cell maintains stable operation for over 1000 h at a current density of 0.5 mA cm^−2^ and an areal capacity of 0.5 mAh cm^−2^. In contrast, lithium symmetric cells with the LE and G-P3 fail at 320 and 767 h, respectively. Additionally, the Li|G-P(CL-U) AR|Li cell experiences a short circuit after cycling for 265 h (Fig. S10), indicating that while the polyester group enhances the ESW, it still suffers from inadequate compatibility with LMAs. This result aligns with the interfacial impedance measurements of symmetric cells after aging for 35 days (Fig. 4c): The interfacial impedance of the Li|G-P3 AR|Li cell increases by only 16% (from 215 to 250 Ω), whereas the Li|LE|Li and Li|G-P3|Li cells exhibit significantly higher growth rates of 46% (120 → 175 Ω) and 58% (314 → 495 Ω). To further evaluate electrolytes’ tolerance for high-capacity lithium plating/stripping, galvanostatic polarization tests were conducted. Figure 4d presents the electrochemical performance of Li||Li symmetric cells with different electrolytes under a current density of 0.1 mA cm^−2^. Before internal short circuiting occurs, the Li|G-P3 AR|Li cell achieves an areal capacity utilization of 53.6 mAh cm^−2^, significantly outperforming the Li|LE|Li cell (20 mAh cm^−2^) and Li|G-P3|Li cell (34 mAh cm^−2^), which demonstrates that the G-P3 AR possesses exceptional lithium hosting capability when interfacing with LMAs.

Previous experiments have demonstrated that the G-P3 AR can optimize solvation structure thereby enhancing the stability of LMAs. The compatibility of electrolytes with high-voltage cathodes is equally critical for practical applications. Thus, LMBs with NCM811 cathodes were assembled and evaluated at 25 °C with a cutoff voltage of 4.3 V. In the rate performance test, specific capacities of 211.2, 202.9, 187.8, 172.9, and 157.7 mAh g^−1^ are achieved at 0.1, 0.2, 0.5, 1 C and 2 C rates for the Li|G-P3 AR|NCM811 cell, respectively. When the rate returns to 0.1 C, the specific capacity recovers to 205.7 mAh g^−1^ (Fig. 4e). Notably, this cell exhibits higher capacities than that of the Li|G-P3|NCM811 cell under all rate conditions. This advantage stems from the enhanced ionic conductivity and *t*_Li+_ provided by DHBA, which accelerates Li^+^ transport kinetics and suppresses the concentration polarization [58, 59]. Furthermore, the Li|G-P3 AR|NCM811 cell shows the highest initial CE (88.65%) (Fig. 4g). The excellent uniformity and passivation ability of the inorganic-rich SEI layer inhibit continuous electrolyte decomposition and the irreversible consumption of lithium sources [60]. When cycled at 0.5 C for charging and 1 C for discharging, the Li|G-P3 AR|NCM811 cell delivers an initial capacity of 176 mAh g^−1^ and maintains 81.7% capacity retention after 500 cycles (Fig. S11), with an average CE > 99.9%. In contrast, the Li|LE|NCM811 and Li|G-P3|NCM811 cells show capacity retentions of 73.5% and 64.7% after 500 cycles, respectively (Fig. 4f). Notably, control cells assembled with single soft segment electrolytes, namely G-P(EG-U) AR (PEG1014 only) and G-P(CL-U) AR (PCL830 only), exhibit markedly inferior cycling stability under identical conditions, corroborating the synergistic effect of combining polyether and polyester segments (Fig. S12). Compared to the performance of the recently reported Li||NCM811 cells, the in situ polymerized polyurethane GPE in this work shows exceptional capacity retention (Fig. 4h, see details in Table S1), further verifying the excellent compatibility of G-P3 AR with both LMAs and high-voltage cathodes. Moreover, to validate the potential application of the designed electrolyte, cells with high-mass-loading NCM811 cathodes (area capacity of 2 mAh cm^−2^) were assembled. The high-mass-loading cell with the G-P3 AR exhibits 76.6% capacity retention after 200 cycles (Fig. S13), whereas the cell with the LE suffers a short circuit as early as 46 cycles. The G-P3 system begins to show significant capacity decay after 118 cycles, with a capacity retention of only 58.6% after 132 cycles. Additionally, the single-layer Li||NCM811 pouch cell with limited lithium supply was assembled using the same cathode. As shown in Fig. S14, the Li|G-P3 AR|NCM811 pouch cell delivers an initial capacity of 193.6 mAh g^−1^ and maintains 85.5% capacity retention over 50 cycles at 0.2 C. Notably, it can power an LED light and remain stable even under bending, puncturing and shearing conditions.

### Interfacial Compatibility

**Fig. 5:**
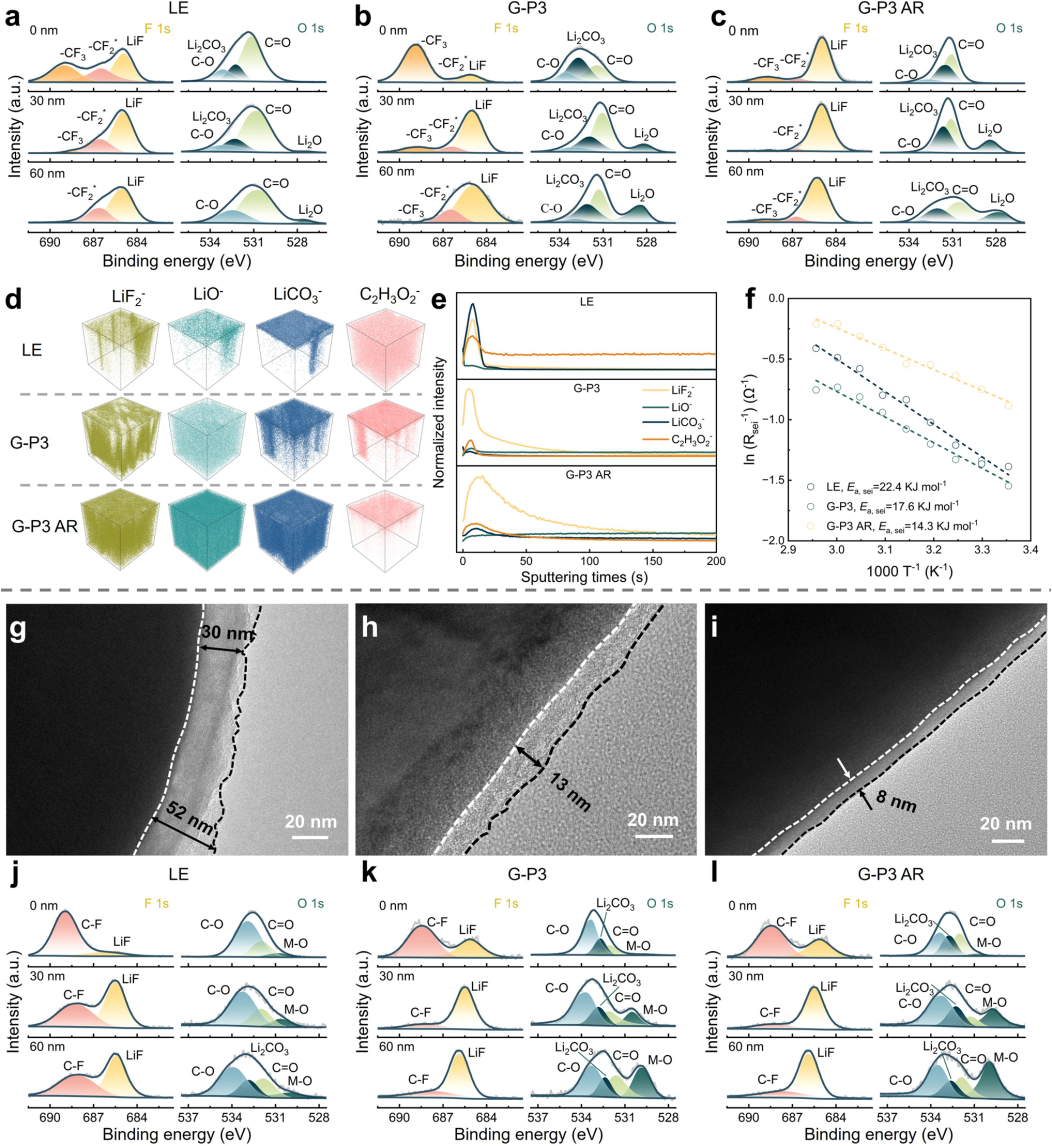
Inorganic-rich SEI/CEI interfacial compatibility mechanism. Depth-profiling F 1*s* and O 1*s* XPS depth spectra of LMAs cycled in Li||Li cells with **a** LE, **b** G-P3 and **c** G-P3 AR. **d** 3D TOF–SIMS mapping images of several representative secondary ion fragments obtained from LMAs with different electrolytes. **e** TOF–SIMS depth profile of different ionic fragments in SEI of different electrolytes. **f** Activation energies of Li^+^ transport through SEI (*E*_a, sei_) derived from *Nyquist* plots. TEM image of NCM811 cathode particles disassembled from cycled Li||NCM811 cells using **g** LE, **h** G-P3 and **i** G-P3 AR electrolytes. Depth-profiling F 1*s* and O 1*s* XPS spectra of NCM811 cathodes cycled in Li||NCM811 cells with **j** LE, **k** G-P3 and **l** G-P3 AR

Scanning electron microscopy (SEM) images reveal that the LE and G-P3 induce mossy lithium deposits and dendritic structures on the lithium surface (Fig. S15a, b), indicating their failure to form a stable SEI capable of suppressing interfacial side reactions and ensuring uniform deposition/dissolution of Li^+^. In contrast, the G-P3 AR enables a smooth and dense lithium metal surface (Fig. S15c), attributed to its low Li^+^ desolvation barrier, which accelerates transport kinetics and reduces solvent-induced interfacial instability. In-depth X-ray photoelectron spectroscopy (XPS) was used to analyze the composition of SEI. As shown in Fig. 5c, F 1*s* and O 1*s* spectra provide crucial insights into the inorganic-rich nature of the SEI formed by the G-P3 AR. In the F 1*s* spectra, the G-P3 AR exhibits a significantly stronger LiF peak at 685 eV compared to those in LE (Fig. 5a) and G-P3 (Fig. 5b), while the –CF_2_* signal at 686.6 eV is substantially attenuated [61, 62], confirming that the LiF-rich SEI layer suppresses further decomposition of lithium salt [63]. The O 1*s* spectra reveal the presence of Li_2_O and Li_2_CO_3_ in the G-P3 AR system, with prominent peaks at 532 eV (Li_2_CO_3_) and 529 eV (Li_2_O) [64]. In contrast, the LE system displays an organically dominated SEI with main C=O (531 eV) and C–O (533 eV) peaks from carbonate solvent decomposition. The C 1*s* spectra serve as supporting evidence, showing a prominent CO_3_^2−^ peak at 290 eV (assigned to Li_2_CO_3_) in the G-P3 AR (Fig. S16) [65], corroborated by the corresponding peaks in the O 1*s* spectra.

Time-of-flight secondary ion mass spectrometry (TOF–SIMS) further verifies the distribution of SEI components. LiF^−^, Li_2_O^−^ and LiCO_3_^−^ ionic fragments are chosen to represent LiF, Li_2_O and Li_2_CO_3_, respectively, and C_2_H_2_O^−^ ionic fragment represents organic component [66]. As shown in Fig. 5d, a high content of LiF, Li_2_CO_3_ and Li_2_O is uniformly distributed on the surface and within the inner layer of SEI for the G-P3 AR cell. Meanwhile, organic components in the SEI are drastically reduced compared to those for the LE and G-P3 systems. The three-dimensional (3D) mapping reveals that inorganic components (LiF, Li_2_O and Li_2_CO_3_) are predominantly distributed throughout the SEI layer. Organic fragments are mainly confined to the surface layer, indicating the formation of an inorganic-rich SEI that provides mechanical strength and ionic conductivity. Figure 5e presents the TOF–SIMS sputtering time-intensity profile, demonstrating the evolution of SEI components with depth. The SEI of the G-P3 AR shows significantly lower organic component signals (C_2_H_2_O^−^) throughout the sputtering process, while maintaining high intensities of inorganic species (especially LiF^−^), which confirms the formation of a robust, inorganic-dominated protective layer. As shown in Figs. 5f and S17, temperature-dependent EIS measurements and calculations of Li||Li cells show that the G-P3 AR has the lowest Li^+^ transport activation energy in SEI (*E*_a, sei_ = 14.3 kJ mol^−1^), versus 17.6 kJ mol^−1^ of the G-P3 and 22.4 kJ mol^−1^ of the LE. This divergence originates from the rapid Li^+^ desolvation process and anion-dominated solvation structure of the G-P3 AR, which endows the SEI with excellent ionic conductivity and thermodynamic stability, further validating its superior interfacial compatibility with LMAs.

To evaluate the electrolyte’s compatibility with high-voltage cathodes, the NCM811 cathodes after 25 cycles were disassembled and characterized. As shown in Fig. 5g, transmission electron microscopy (TEM) images reveal that the CEI layer on the NCM811 cathode using the LE exhibits a non-uniform thickness ranging from 30 to 52 nm. In contrast, the introduction of in situ polymerized polyurethane leads to a uniform CEI layer with obviously reduced thickness (Fig. 5h, i). Notably, the CEI layer of Li|G-P3 AR|NCM811 cell displays the most compact and uniform morphology, with a thickness of only 8 nm. Subsequently, XPS depth profiling was employed to characterize the CEI components. As shown in Fig. 5j–l, the analysis of F 1*s* spectra demonstrates a more intense inorganic LiF signal on the surface of the G-P3 AR-cycled cathode compared to the LE and G-P3 systems (the C–F signal primarily originates from the PVDF binder [67]). The O 1*s* and C 1*s* spectra indicate that the intensity of Li_2_CO_3_ peak for the cathode coupled with polyurethane gel electrolyte is significantly higher than that of the Li|LE|NCM811 cell, while the surface of the LE-cycled cathode shows stronger signals of organic products (Figs. 5j–l and S18). Furthermore, the intensity of the metal (M) –O peak serves as an indicator of CEI thickness. Its significantly suppressed intensity provides direct evidence for the formation of thicker CEI layer on the LE-cycled cathode, which prevents the effective probing of the interior NCM811 by XPS. These results indicate that the solvation reconstruction effect of the G-P3 AR promotes the formation of robust, dense SEI and CEI layers, thereby demonstrating the superior interfacial compatibility with both LMAs and NCM811 cathodes.

## Conclusions

Based on the synergistic modulation of atomic and molecular structures, this work successfully develops an in situ cross-linked polyurethane gel electrolyte (G-P3 AR) for high-performance LMBs. It achieves an extended ESW of 4.97 V, a high *t*_Li+_ of 0.78, and excellent compatibility with both LMAs and high-voltage NCM811 cathodes. The rational molecular design incorporates polyester segments to enhance oxidation resistance, polyether segments to facilitate rapid desolvation, and *sp*^2^-hybridized boron atoms to immobilize anions via Lewis acid–base interactions. Furthermore, the abundant hydrogen bonding restructures the solvation sheath, promoting anion participation and the formation of inorganic-rich SEI/CEI layers. As a result, the Li||Li symmetric cell demonstrates stable cycling over 1000 h, and the Li||NCM811 coin cell retains 81.7% capacity after 500 cycles at 0.5 C charge/1 C discharge. This work provides a scalable and practical electrolyte design paradigm, offering fundamental insights into structure-performance relationships for next-generation energy storage systems.

## Supplementary Information

Below is the link to the electronic supplementary material.

Supplementary file1 (DOCX 4269 KB)
