# Supplementary material for: Atomic and Molecular Structure Regulated In Situ Cross-Linked Polyurethane Gel Electrolyte for High-Performance Lithium Metal Batteries
Source: Nanomicro Lett. 2026 Aug 3;19:18. doi: 10.1007/s40820-026-02307-4 (PMC13433712; doi:10.1007/s40820-026-02307-4)
Supplement: Supplementary file 1 [file 40820_2026_2307_MOESM1_ESM.docx]

Supporting Information for

**Atomic and Molecular Structure Regulated In Situ Cross-Linked Polyurethane Gel Electrolyte for High-Performance Lithium Metal Batteries**

Jialun Ni ^1, +^, Yong Zeng ^2, +^, De Ning ^3^, Xuan He ^4^, Xiaokang Ju ^4^, Xueling Liu ^1^, Rui Gao ^1^, Yingchun Xu ^2^, Ruijie Du ^1^, Dong Zhou ^5,^ ^*^, Jun Wang ^2, *^ and Yongli Li ^1,^ *

^1^ Institute for Clean Energy Technology, North China Electric Power University, Beijing 102206, P. R. China

^2^ School of Innovation and Entrepreneurship, Southern University of Science and Technology, Shenzhen 518055, P. R. China

^3^ Centre for Photonics Information and Energy Materials, Shenzhen Institutes of Advanced Technology, Chinese Academy of Sciences, Shenzhen 518055, P. R. China

^4^ Sunwoda Mobility Energy Technology Co., Ltd, Shenzhen 518107, P. R. China

^5^ School of Advanced Energy, Sun Yat-sen University Shenzhen Campus, Shenzhen 518107, P. R. China

^+^ Jialun Ni and Yong Zeng contributed equally to this work.

*Corresponding authors. E-mail: [zhoud37@mail.sysu.edu.cn](mailto:zhoud37@mail.sysu.edu.cn) (Dong Zhou); [wangj9@sustech.edu.cn](mailto:wangj9@sustech.edu.cn) (Jun Wang); [yongli.li@ncepu.edu.cn](mailto:yongli.li@ncepu.edu.cn) (Yongli Li)

**S1 Theoretical Calculation**

MD: The molecular dynamics (MD) were performed using the software package GROMACS (version 2021.3). After Gaussian16 generated all optimized molecules, a system was constructed using packmol consisting of specific molecules enclosed within a cubic box with an edge size of 5 nm. The atomic interactions were parameterized by the optimized potentials for liquid simulations all-atom (OPLS-AA) force field, and RESP2 charge obtained from Multiwfn was applied in the calculations. After the energy minimization, the systems were pre-balanced in NPT ensemble with Berendsen method for 10 ns followed by a production run in the NVT ensemble at 300 K with a time step of 1 fs. The temperature of the system was regulated by a nose-hoover thermostat (τT = 1 ps). After 25 ns of simulation, the mean square displacement (MSD) and radial distribution function (RDF) of the particles were analyzed by the GROMACS toolkit.

DFT: The molecular geometries for the ground states were optimized by density functional theory calculation at the B3LYP/6-311+G* level, and then the HOMO, LUMO and the molecular electrostatic potential were performed with the GAUSSIAN 16 software package with a basis set of 6-311+G*. The optimized structures were confirmed to be true minima by analytical vibrational frequency calculations.

**S****2 Supplementary Figures**

**Fig. S1** **a** Electron cloud density distribution and charge distribution of the DHBA-centered polyurethane segment. Electron cloud density distribution and binding energy of **b** DHBA-centered polyurethane segment with TFSI^–^ and **c** DHBA-centered polyurethane segment with DFOB^–^

**Fig. S2** Optical photos of **a** G-P3, **b** G-P3 AR before and after polymerization. **c** FTIR spectra of small molecular monomers and GPEs. **d** FTIR spectra of GPEs

**Fig. S3** Optical photos of **a** G-P3 precursor without DBTDL, **b** G-P3 AR precursor without DBTDL before and after heating. **c** FTIR spectra of G-P3, G-P3 AR and the corresponding precursors without DBTDL after heating

**Fig. S4** *Nyquist* plots of **a** LE, **b** G-P3 and **c** G-P3 AR at different temperatures

**Fig. S5 a** XRD patterns and **b** DSC curves of P3 and P3 AR

**Fig. S6** LSV curves of G-P(EG-U), G-P(CL-U) and G-P3

**Fig. S7** Polarization curves and the *Nyquist* plots before and after polarization of **a** Li|LE|Li and **b** Li|G-P3|Li

**Fig. S8** FTIR spectra of **a** P3 AR and G-P3 AR, **b** P3and G-P3

**Fig. S9** Li plating/stripping curves of Li||Cu half cells with **a** LE and **b** G-P3

**Fig. S10** Li plating/stripping curves of Li|G-P(CL-U) AR|Li cell

**Fig. S11** Galvanostatic charge/discharge curves of Li|G-P3 AR|NCM811 (3 mg cm^−2^) cell in the voltage range of 3.0–4.3 V using 0.5C charge/1C discharge at different cycles

**Fig. S12** Cycling performance of Li||NCM811 cells with different electrolytes at 0.5C charge/1C discharge

**Fig. S13** Cycling performance of Li||NCM811 (10 mg cm^−2^) cells with different electrolytes in the voltage range of 3.0–4.3 V at 0.2C charge/discharge

**Fig. S14 a** Cycling performance of Li|G-P3 AR|NCM811 pouch cell in the voltage range of 3.0–4.3 V at 0.2C charge/discharge. **b** Illustrations of Li|G-P3 AR|NCM811 pouch cell lightening experiments under extreme conditions

**Fig. S15** Surface SEM images of LMAs disassembled from cycled Li||Li cells with **a** LE, **b** G-P3 and **c** G-P3 AR. Insets: Corresponding photo images

**Fig. S16** C 1s XPS depth profiling results of LMAs cycled in Li||Li cells with **a** LE, **b** G-P3 and **c** G-P3 AR

**Fig. S17** *Nyquist* plots of Li||Li cells with LE, G-P3 and G-P3 AR at different temperatures

**Fig. S18** C 1s XPS depth profiling results of NCM811 cathodes cycled in Li||NCM811 cells with **a** LE, **b** G-P3 and **c** G-P3 AR

**S3 Supplementary Table**

**Table S1** Comparison of cell performances of this work with typical GPEs and QSEs in literature. For the cells listed in this table, Li metal was used as anode, and NCM811 was used as cathode. All measurements were performed at room temperature

| **Electrolytes** | **Journal and year of publication** | **Performance** | **Capacity lost per cycle/%** | **Ref.** |
| --- | --- | --- | --- | --- |
| PFVS | Adv. Energy Mater. 2024 | 100 cycles, 81.5% retention, 0.25 mA cm^−2^ | 0.203 | [S1] |
| WSGPE | Nat. Commun. 2025 | 200 cycles, 90.8% retention, 0.6 mA cm^−2^ | 0.048 | [S2] |
| MPDE | Energy Environ. Sci. 2025 | 100 cycles, 87.3% retention, 0.1 mA cm^−2^ | 0.137 | [S3] |
| L-AGPE | Energy Environ. Sci. 2025 | 500 cycles, 70.5% retention,  0.5 mA cm^−2^ | 0.07 | [S4] |
| E-QSPE | Energy Storage Mater. 2024 | 350 cycles, 80% retention,  0.2 mA cm^−2^ | 0.063 | [S5] |
| NGPE | Nat. Energy. 2023 | 500 cycles, 75% retention,  0.3 mA cm^−2^ | 0.057 | [S6] |
| F-PIL | Adv. Energy Mater. 2025 | 240 cycles, 80% retention,  ~ 0.25 mA cm^−2^ | 0.093 | [S7] |
| PDA@CityU-43 | Angew. Chem. Int. Ed. 2025 | 100 cycles, 72.5% retention,  0.116 mA cm^−2^ | 0.319 | [S8] |
| TPDOL | Adv. Mater. 2024 | 150 cycles, 85.6% retention,  0.66 mA cm^−2^ | 0.102 | [S9] |
| FE18N | Adv. Energy Mater. 2024 | 300 cycles, 79.2% retention,  0.3 mA cm^−2^ | 0.075 | [S10] |
| **This work** |  | 500 cycles, 81.7% retention,  0.6 mA cm^−2^ | **0.041** |  |

**Supplementary References**

[S1]	H. Peng, T. Long, J. Peng, H. Chen, L. Ji et al., Molecular Design for In-Situ Polymerized Solid Polymer Electrolytes Enabling Stable Cycling of Lithium Metal Batteries. Adv. Energy Mater. **14**(22), 2400428 (2024). <https://doi.org/10.1002/aenm.202400428>

[S2] S. Liu, W. Tian, J. Shen, Z. Wang, H. Pan et al., Bioinspired gel polymer electrolyte for wide temperature lithium metal battery. Nat. Commun. **16**, 2474 (2025). <https://doi.org/10.1038/s41467-025-57856-w>

[S3]	H. Xu, J. Mi, J. Ma, Z. Han, S. Lv et al., Mg^2+^ initiated in situ polymerization of dioxolane enabling stable interfaces in solid-state lithium metal batteries. Energy Environ. Sci. **18**(9), 4231-4240 (2025). <https://doi.org/10.1039/D4EE05606J>

[S4] H. Yang, J. Yan, S. Gao, X. Chen, Y. Wang et al., An asymmetric functional gel polymer electrolyte enables superior interfacial compatibility for wide temperature lithium metal batteries. Energy Environ. Sci. **18**(22), 9854-9864 (2025). <https://doi.org/10.1039/D5EE03838C>

[S5] Y. He, X. Shan, Y. Li, Z. Li, L. Li et al., In-situ formation of quasi-solid polymer electrolyte for wide-temperature applicable Li-metal batteries. Energy Storage Mater. **68**, 103281 (2024). <https://doi.org/10.1016/j.ensm.2024.103281>

[S6] Y. Meng, D. Zhou, R. Liu, Y. Tian, Y. Gao et al., Designing phosphazene-derivative electrolyte matrices to enable high-voltage lithium metal batteries for extreme working conditions. Nat. Energy **8**, 1023-1033 (2023). <https://doi.org/10.1038/s41560-023-01339-z>

[S7]	K. Li, A. Hu, R. Xu, W. Xu, B. Yang et al., Decoupled Ion Transport via Triadic Molecular Synergy in Flame-Retardant Quasi-Solid Electrolytes for Safe Lithium Metal Batteries. Adv. Energy Mater. **15**(28), 2501236 (2025). <https://doi.org/10.1002/aenm.202501236>

[S8] T. Naren, Q. Gu, R. Jiang, Y. Zhao, L. Zhang et al., Nanoconfined Polymerization Facilitates Efficient Li+ Transportation in Quasi-Solid Electrolytes. Angew. Chem. Int. Ed. **64**(33), e202509921 (2025). <https://doi.org/10.1002/anie.202509921>

[S9]	S.-J. Yang, H. Yuan, N. Yao, J.-K. Hu, X.-L. Wang et al., Intrinsically Safe Lithium Metal Batteries Enabled by Thermo-Electrochemical Compatible In Situ Polymerized Solid-State Electrolytes. Adv. Mater. **36**(35), 2405086 (2024). <https://doi.org/10.1002/adma.202405086>

[S10] C. Zhao, Y. Lu, K. Yan, Y. Guan, S. Jiang et al., Tailoring the Chemical/Electrochemical Response in a Quasi−Solid Polymer Electrolyte Enables the Simultaneous In Situ Construction of Superior Cathodic and Anodic Interfaces. Adv. Energy Mater. **14**(22), 2304532 (2024). <https://doi.org/10.1002/aenm.202304532>
